# Cochlear implant for profound hearing loss post COVID-19 complications^☆^

**DOI:** 10.1016/j.bjorl.2023.101320

**Published:** 2023-09-04

**Authors:** Amanda Sampaio Almeida, Fernando Kaoru Yonamine, Alexandra Dezani Soares, Norma de Oliveira Penido, Oswaldo Laercio Mendonça Cruz

**Affiliations:** Universidade Federal de São Paulo (UNIFESP), Escola Paulista de Medicina, Departamento de Otorrinolaringologia e Cirurgia de Cabeça e Pescoço, São Paulo, SP, Brazil

## Abstract

• COVID-19 has been associated to Sensorineural Hearing Loss.
• Cochlear implants may benefit patients with profound hearing loss post COVID-19.
• Hearing rehabilitation should not be postponed in cases of deafness post COVID-19.

## Introduction

The effects of the Coronavirus SARS-Cov-2 (COVID-19) have been related to different clinical presentations, unpredictable evolutions, and outcomes. Otological and neurological complaints have also been associated with COVID-19, including deafness.^1^ As with other viruses, the pattern is a Sensorineural Hearing Loss (SNHL) from mild to profound.

In cases of severe to profound SNHL, Cochlear Implant (CI) would be indicated, however, surgical implications and performance in COVID-patients have yet to be studied.^2^ We present a case of a patient with bilateral profound SNHL post-COVID-19 who underwent simultaneous bilateral CI surgery.

## Case report

A 55-year-old male with a past medical history of hypertension, hypothyroidism and anxiety presented symptoms described as cough, headache, and fever on March 11st 2021. He was positive for SARS-CoV-2 and did not clinically improve with analgesics. On March 19th he was admitted in Intensive Unit Care (IUC) for acute respiratory syndrome.

During hospitalization, he was intubated and had acute renal failure, undergoing mechanical ventilation and hemodialysis. Cefepime was associated with vancomycin. Ten days later, he presented worsening hemodynamics conditions. Antibiotics had been staggered to Meropenem and a tracheostomy was performed. One month later, he presented a convulsive crisis status witnessed in electroencephalogram and new cultures showed Pseudomonas, treated with Piperacillin Sodium-Tazobactam.

On May 12th, he started daily interruption of sedatives and eight days later, he complained of bilateral hearing loss. An audiogram showed no response and Brain Evoked Response Audiometry (BERA) confirmed bilateral profound SNHL. After 98 days hospitalized, he was discharged to a rehabilitation center. Until that moment, patient had no previous vaccination for COVID-19.

On March 2022 he was referred to our center, complaining of bilateral anacusis and a sporadic tinnitus described as “machine noise”. His otoscopy and Computed Tomography (CT) were normal. Magnetic Resonance Imaging (MRI) showed increased signal on T2 and FLAIR in the periventricular white matter suggesting alteration of microangiopathy nature.

Audiometric test has showed a profound SNHL in both ears. Speech Reception Threshold (SRT) could not be defined. Tympanograms were normal and acoustic reflexes were absent. Speech perception test was 0% in open-set and closed-set. Transiently evoked and distortion-product otoacoustic emissions were absent.

Bilateral cochlear implantation was performed with a Nucleus CI622 device. During surgery, it was noted middle ear mucosal hyperplasia, without any effusion. Full insertion of electrodes array occurred without complications and no sign of cochlea ossification. Impedance field telemetry determined integrity bilaterally.

During initial activation, patient reported reasonable hearing and improving of tinnitus. Postoperative pure-tone audiometry two months after activation showed improvement of SRT, mainly with both implants (Table 1). Patient achieved 88% intelligibility of disyllables and 64% of monosyllables at 65 dB with both CI. He scored 96% on sentence recognition on silence at 65 dB (Table 2). Currently, he continuously reports improvement on understanding and tinnitus.

**Table 1:** Postoperative pure-tone audiometry 2 months after CI activation.

	250 Hz	500 Hz	1000 Hz	2000 Hz	3000 Hz	4000Hz	6000 Hz
CI RE	35 dB	25 dB	50 dB	50 dB	35 dB	30 dB	25 dB
CI LE	40 dB	50 dB	35 dB	35 dB	45 dB	40 dB	20 dB
CI RE+LE	30 dB	35 dB	35 dB	30 dB	30 dB	30 dB	20 dB

CI, Cochlear Implant; LE, Left Ear; RE, Right Ear.

**Table 2:** Postoperative Speech Perception 2 months after CI activation.

	Live Speech				Recorded Speech	
	SRT	Mono	Dis	Silence	Mono	Silence
CI RE	35 dB	28%	72%	96%	04%	86%
CI LE	40 dB	44%	68%	96%	16%	85.71%
CI RE+LE	30 dB	64%	88%	96%	16%	100%

CI, Cochlear Implant; LE, Left Ear; Mono, Monosyllables; Dis, Disyllables; RE, Right Ear; SRT, Speech Recognition Threshold.

Patient consent and Universidade Federal de São Paulo Institutional Review Board approval were obtained under number 1466/2020.

## Discussion

Hearing loss related to COVID-19 has been reported worldwide. However, there are a restricted number of cases with indication for Cochlear Implant (CI) and few studies that present its outcomes in this specific population.

The pathophysiology of COVID-related SNHL is not completely understood and degree and duration of this sequel is still unpredictable. Furthermore, the association between SNHL and SARS-CoV-2 can be biased by confounding factors. Assuming a direct cochlear involvement, with ossification and fibrosis, CI surgery should not be postponed in order to insert the electrode array without complications.^1^ Such urgency was noted in one described case,^3^ with MRI suggesting inflammatory process in the cochlea. Another theory would be thromboembolism and the vasculitis^4^ probably linked to increased Angiotensin-Converting Enzyme 2 (ACE2) and an excess of cytokines. In our patient, MRI showed signs of microangiopathy, which leads us to suspect a multifactorial cause: vascular aggression to the vestibulocochlear nerve and possible effect of Vancomycin. It is observed that in most patients with post-Covid deafness, it is a multiple-factorial condition.

In case of permanent neurological damage caused by COVID-19, a great CI performance would not be expected. A report of two patients^5^ observed a decrease in auditory performance and speech comprehension in two previously implanted children after being affected by the coronavirus. Oppositely, there are two published reports of post-covid implanted patients who also had satisfactory outcomes.^1^, ^2^

## Conclusion

It is consensus that patients with acute symptoms, including SNHL, should be promptly tested for COVID-19. While research is being published, audiological rehabilitation should not be postponed, including CI in cases of profound SNHL.

## Authors’ contributions

Amanda Sampaio Almeida: Surgical procedure, literature review, data collection, study’s design and writing.

Fernando Yonamine: Surgical procedures and study’s writing.

Alexandra Dezani Soares: Data collection and study’s writing.

Norma de Oliveira Penido: Literature review and study’s writing.

Oswaldo Laercio Mendonça Cruz: Literature review, study’s design and writing.

All authors read and approved the final manuscript.

## Conflicts of interest

The authors declare no conflicts of interest.
